# Scientific Items

**Published:** 1885-05-20

**Authors:** 


### SCIENTIFIC ITEMS.

**Petroleum in Russia.**—The Russian oil region covers an area of over 14,000 square miles, with forty-two oil wells in one district, over a hundred in another, four hundred in a third, and richer regions waiting to be developed to produce still greater results. One spouting well produces, it is said, two million of gallons a day. The oil is found in places at a depth of a hundred feet, and no well has gone below eight hundred and twenty-five feet. Three Swedish brothers, and a few others, English and Americans, as well as Russians, who have been in America, have introduced method and system, pipe lines, oil carrying barges and steamers, tank cars, refineries, joint stock companies, railroads, and now produce 800,000 tons of crude and 200,000 tons of refined petroleum, and are rapidly finding new markets. In America there are over 25,000 drilled petroleum wells; in Baku, the Russian oil region of most activity, there are 400, but a single one of these, it is claimed, has thrown up as much oil in a day as nearly the whole of the 20,000 in America put together.

Spouting wells in Russia are both frequent and constant, and the overflow is sometimes a serious difficulty, in some cases run into the sea or low land, and burned to get rid of it.—*Scientific American.*

**Feat of the Divining Rod.**—The question as to the magical or the scientific value of the "divining rod" has just been re-opened by the success which has attended its use at the Fletton Wagon Works of the Midland Railway Company, England, with reference to the discovery of a permanent supply of water. According to *The Sanitary World* (London), the company requires to use about 500 or 600 gallons of water every day, and the well on their premises yielded only one-half of that quantity. It was necessary, therefore, to supplement the supply by the sinking of other wells or by the construction of an expensive system of piping. The former plan was preferred, and two new wells were sunk to no purpose. The services of a gentleman of the district, who bore the reputation of being skilled in the art of discovering water by means of the "divining rod," were then called in. This wizard or expert employed for his purpose a forked hazel twig, holding one prong of the fork in each hand, the points of the fork being directed to the sky. After walking about the premises for some time, the point of the fork suddenly began to bend down, purely, as the best evidence goes, of its own accord, and to point to the earth. The wielder of the wand declared that here would be found a plentiful supply of water. The same indications were repeated at another spot, where the twig snapped from the violence of its spontaneous and sympathetic motion, and the same confident assertions were made with reference to the occurrence of water—

assertions which the results obtained by actually sinking wells amply justified, the quantity of water to be obtained being apparently inexhaustable. Other persons essayed to use the wand, but it rebelled against the usurpation of its owner's functions, and remained contumacious and irresponsive. If any person, adds the writer, require water in unlucky localities, it might be well to secure the services of this diviner before he volunteers for a patriotic mission in favor of the troops in the thirsty wilds of the Soudan.—*Scientific American.*

**The Painless Extinction of Life of Animals.**—Dr. Richardson, of London, in a lecture on his process of painless killing of the lower animals, said in the closing passages of his discourse at the Dogs' Home over 6,000 dogs have during the past seven months slept their final sleep, knowing as little of their deaths as of their births. The principal agent used for the narcotic action is carbonic oxide, passing, at summer heat, over a mixture of chloroform and carbon bisulphide into a lethal chamber, in which chamber as many as 100 dogs can at once receive euthanasia. This is on the large scale; but Dr. Richardson described also a small apparatus in which from one to six animals can be painlessly killed, and which is so portable that it can be wheeled from a central station to any house or street ready for immediate use. Thus every village and town may be provided at a small cost with a means that will give painless death to any domestic animal without offending the most sensitive individual. By an extension of the same design the author next intends to apply it to animals of the larger kind that are used for human food. It is no contemptible part of its history in this century for the profession to leave as a bequest to the future, the means of taking the sting of death from all the lower animals whose fate is under our control.—*Scientific American.*

**At the Top of Mount Washington.**—A visitor to the top of Mount Washington concludes that the weather is really cold up there. He was convinced by a walk along the railroad, with the wind blowing seventy miles an hour and the thermometer twenty degrees below zero. The temperature does not get lower there than in many other places, but the wind blows with greater velocity, it is said, than at any known spot in the world, and this makes the cold unbearable. A velocity of 180 miles an hour has been attained, while at Pike's Peak, 8,000 feet higher, the greatest is 100 miles, and in New York 45 miles is a heavy gale.

The cold is so intense that if one covers every part of the body, leaving only the eyes exposed, these are soon coated with frost, which closes the lids and often makes it almost impossible to see. The moisture of the breath freezes under the covering of the face, and a frost bite is the consequence.—*Scientific American*

**Baron von Schoeler**, of Corpus Christi, has for a pet an immense snake of the anaconda species. It is perfectly docile so far as the Baron has yet learned.—*Ibid.*

